# Comment on Vázquez-Gandullo et al. Inspiratory Muscle Training in Patients with Chronic Obstructive Pulmonary Disease (COPD) as Part of a Respiratory Rehabilitation Program Implementation of Mechanical Devices: A Systematic Review. *Int. J. Environ. Res. Public Health* 2022, *19*, 5564

**DOI:** 10.3390/ijerph20054629

**Published:** 2023-03-06

**Authors:** Safak Yigit, Buket Akinci

**Affiliations:** 1 Department of Therapy and Rehabilitation, Vocational School, Istanbul Galata University, Istanbul 34425, Turkey; 2 Department of Physiotherapy and Rehabilitation, Faculty of Health Sciences, Biruni University, Istanbul 34010, Turkey

In May 2022, the paper “Inspiratory Muscle Training in Patients with Chronic Obstructive Pulmonary Disease (COPD) as Part of a Respiratory Rehabilitation Program Implementation of Mechanical Devices: A Systematic Review” was published in the *International Journal of Environmental Research and Public Health*. Sixteen studies were included in the review, and it was concluded that in the pulmonary rehabilitation program in which the inspiratory muscles were trained by using the Inspiratory Muscle Training (IMT) devices benefited the quality of life in patients with COPD [1]. We have read with great interest the systematic review which gives broad information about types of IMT and current devices used for the training. We would like to share our views and ask questions about the review.

First, the authors stated that studies with low quality evidence were excluded from the review. According to the PRISMA guideline, most methods for assessing the quality of studies to be included in the systematic review include components such as the suitability of the research design, the risk of bias, the choice of measurement method, and the quality of the intervention administered [2,3]. However, we could not read about a cut-off point for the quality of the studies or information about quality assessment in this review. Therefore, it is difficult to discuss the quality, integrity and homogeneity of the included studies.

Secondly, in the training of respiratory muscles, the principles of loading, specialization and reversibility are taken into account, as in peripheral muscles. To provide response in inspiratory muscle training, muscle fibers must be loaded. For the loading principle of respiratory muscle training, the training should be applied at a certain intensity, duration and frequency [4]. Although device-specific information is given in detail for the 16 studies, enough information is not provided about the usage regime of IMT protocol. As a result of the review, do authors suggest a specific IMT protocol for the COPD patients? Additionally, which type of training is better for COPD patients in terms of patient compliance?

Finally, a systematic review and meta-analysis conducted in 2018 examined the effects of inspiratory muscle training in COPD patients. The review included 37 studies in the meta-analysis which all evaluated quality of life, and it was reported that IMT using threshold improves quality of life [5]. In the current review, only three out of the 16 included studies evaluated quality of life; however, the authors concluded that IMT devices provided a wide benefit in terms of quality of life. Can we think that the increased quality of life is a result of other clinical improvements achieved with the IMT, or was the methodological quality of these three studies significantly high?

There is increasing evidence of the benefits of IMT even applied alone for patients with COPD [6]. Therefore, we believe that this review is an important guide for clinicians, and further suggestions according to our questions can bring us a different perspective on the rehabilitation program of patients with COPD. We look forward to your answers and thank you in advance.
